# Economic evaluations of radioembolization with Itrium-90 microspheres in hepatocellular carcinoma: a systematic review

**DOI:** 10.1186/s12876-022-02396-6

**Published:** 2022-07-02

**Authors:** J. C. Alonso, I. Casans, F. M. González, D. Fuster, A. Rodríguez, N. Sánchez, I. Oyagüez, R. Burgos, A. O. Williams, N. Espinoza

**Affiliations:** 1 Nuclear Medicine Department, Hospital Gregorio Marañón, Madrid, Spain; 2 Nuclear Medicine Department, Hospital Clínico Universitario, Valencia, Spain; 3 Nuclear Medicine Department, Hospital Universitario Central, Asturias, Spain; 4 Nuclear Medicine Department, Hospital Clinic, Barcelona, Spain; 5 Nuclear Medicine Department, Hospital Virgen de las Nieves, Granada, Spain; 6 Pharmacoeconomics & Outcomes Research Iberia (PORIB), P. Joaquín Rodrigo 4 - letra I, 28224 Pozuelo de Alarcón, Madrid Spain; 7 Boston Scientific Iberia, Madrid, Spain; 8 Boston Scientific, Marlborough, MA USA

**Keywords:** Carcinoma, Hepatocellular, Liver neoplasms, Radiotherapy, Yttrium-90, Cost, Systematic review

## Abstract

**Background:**

Transarterial radioembolization (TARE) with yttrium-90 microspheres is a clinically effective therapy for hepatocellular carcinoma (HCC) treatment. This study aimed to perform a systematic review of the available economic evaluations of TARE for the treatment of HCC.

**Methods:**

The Preferred Reported Items for Systematic reviews and Meta-Analyses guidelines was followed by applying a search strategy across six databases. All studies identified as economic evaluations with TARE for HCC treatment in English or Spanish language were considered. Costs were adjusted using the 2020 US dollars based on purchasing-power-parity ($US PPP).

**Results:**

Among 423 records screened, 20 studies (6 cost-analyses, 3 budget-impact-analyses, 2 cost-effectiveness-analyses, 8 cost-utility-analyses, and 1 cost-minimization analysis) met the pre-defined criteria for inclusion. Thirteen studies were published from the European perspective, six from the United States, and one from the Canadian perspectives. The assessed populations included early- (n = 4), and intermediate-advanced-stages patients (n = 15). Included studies were evaluated from a payer perspective (n = 20) and included both payer and social perspective (n = 2). TARE was compared with transarterial chemoembolization (TACE) in nine studies or sorafenib (n = 11). The life-years gained (LYG) differed by comparator: TARE versus TACE (range: 1.3 to 3.1), and TARE versus sorafenib (range: 1.1 to 2.53). Of the 20 studies, TARE was associated with lower treatment costs in ten studies. The cost of TARE treatment varied widely according to Barcelona Clinic Liver Cancer (BCLC) staging system and ranged from 1311 $US PPP/month (BCLC-A) to 71,890 $US PPP/5-years time horizon (BCLC-C). The incremental cost-utility ratio for TARE versus TACE resulted in a 17,397 $US PPP/Quality-adjusted-Life-Years (QALY), and for TARE versus sorafenib ranged from dominant (more effectiveness and lower cost) to 3363 $US PPP/QALY.

**Conclusions:**

Economic evaluations of TARE for HCC treatment are heterogeneous. Overall, TARE is a cost-effective short- and long-term therapy for the treatment of intermediate-advanced HCC.

**Supplementary Information:**

The online version contains supplementary material available at 10.1186/s12876-022-02396-6.

## Background

Hepatocellular carcinoma (HCC) is the most common type of primary neoplasm of the liver, the sixth most common cancer, and the third leading cause of cancer death globally [1–3]. Liver cancer mortality accounts for 8.4% of all cancer deaths as of 2020 [3]. Patients with HCC have a significant humanistic and economic burden [4]. The annual direct costs for HCC patients, regardless of stage or treatment, ranged from $29,354.47 to $58,529.45 per patient in the United States. Also, indirect costs, such as reduced labour productivity, account for 10.8% ($49.1 million) of the overall annual cost (direct and indirect) of HCC [4].

The Barcelona Clinic Liver Cancer (BCLC) staging system is the most widely used and most frequently recommended by scientific societies. This is the only system that relates the prognostic evaluation (based on 5 stages) to the different treatment options [1, 2]. The recently updated BCLC guideline recommends first-line treatments such as ablation, resection, transplantation, and transarterial radioembolization (TARE) as an option for patients in the early stages of the disease (BCLC-0, BCLC-A) or patients with a tumour size ≤ 8 cm who are not eligible for ablative techniques or resection. For the intermediate stage (BCLC-B), treatment options include transplantation for patients with well-defined nodules, transarterial chemoembolization (TACE) for patients with the preserved portal flow, and a defined tumour burden, or systemic therapy. For advanced-stage (BCLC-C), systemic therapy based on immunotherapy (a combination of atezolizumab and bevacizumab) is the main treatment option, and the second line option is tyrosine kinase inhibitors (TKIs). The treatment option in the terminal stage (BCLC-D) is palliative care [2].

The characteristics of the predominant arterial flow in patients with HCC have justified treatment with intra-arterial therapies, such as TARE with yttrium 90 microspheres (^90^Y-TARE) as a therapeutic option for HCC. ^90^Y-TARE has demonstrated clinical efficacy as an alternative treatment for HCC in radiological response and shown adequate safety profile in patients in different stages of the disease [2]. In the early to intermediate stage of HCC, treatment with TARE prolongs the time to progression, which reduces the withdrawal from transplant or surgical resection waiting lists [5, 6]. In the advanced stage of HCC, available evidence (the SARAH [7] and SIRveNIB [8] studies) has determined ^90^Y-TARE presents an efficacy profile and survival benefit compared to sorafenib. Also, when the combination of ^90^Y-TARE with sorafenib was evaluated (the SORAMIC study [9]), the toxicity was no greater than sorafenib monotherapy [9].

A recent update of the European Society of Medical Oncology (ESMO) clinical practice guidelines recommends using ^90^Y-TARE as an alternative treatment in the early and intermediate stages of HCC. The guideline recommends using TARE in exceptional circumstances, patients with diseases limited to the liver or with a good liver function but for whom TACE or systemic therapy is not possible [10]. Two types of microspheres are known to include the beta ^90^Y emitter: glass (TheraSphere^®^) [11] and resin (SIR-Spheres^®^) microspheres [12]. Additionally, there is a third type based on holmium-166 (^166^Ho, QuiremSpheres^®^) [13] that was not included in the review due to limited clinical evidence, as indicated by the National Institute for Clinical Excellence (NICE) [14].

In addition to the clinical evidence, economic studies justify the use of new innovative therapies to optimize clinical outcomes in the context of the National Health System (NHS). Given the clinical benefits, limited economic resources, and greater emphasis placed on strengthening healthcare systems, there is an inherent need to generate economic evidence that enhances efficiency and prioritizes the available health resources [15]. Subsequently, a review of the economic benefits of ^90^Y-TARE in the HCC population needs to be established. Thus, this systematic review aimed to review and summarize the economic evaluations of the use of ^90^Y-TARE for the treatment of primary hepatic neoplasms, specifically HCC.

## Methods

### Search strategy and identification of studies

A systematic review of all economic evaluations on TARE for the treatment of HCC and published in Spanish and English was conducted following the Preferred Reporting Items for Systematic Reviews and Meta-Analyses (PRISMA) methodology [16, 17].

The search strategy was designed using the Population, Intervention, Comparison, Outcomes (PICO) methodology. Also, Boolean operators without limitations and by these criteria: type of study, language, or year of publication (except the limitation of the search of communications to congresses to a 5-year period) were applied. A manual search of the citations of the initially selected articles was performed to identify potentially relevant additional publications. Key search terms included “Hepatocarcinoma”, “Hepatic neoplasms”, “Primary liver tumour”, “Primary liver tumours”, “Liver metastases”, “Secondary liver cancer”, “Hepatocellular carcinoma”, “HCC”, “Intrahepatic cholangiocarcinoma”, “Colorectal metastasis”, “Colorectal metastases”, “Colorectal carcinoma”, “Colorectal neoplasms”, “Colon”, “Neuroendocrine tumours”, “Yttrium-90”, “90Y”, “90-Y”, “Y-90”, “Y90”, “radioembolization”, “transarterial radioembolization”, “transcatheter arterial radioembolization”, “TARE”, “Selective internal radiation therapy”, “SIRT”, “sirtuins”, “TheraSphere”, “SIR-Spheres”, “SIRSpheres”, “Cost”, “Cost utility”, “Cost benefit”, “Cost efficiency”, “Cost analysis”, “Budget impact” and “economic evaluation” (Additional file 1).

Databases were searched for all economic evaluations using ^90^Y-TARE for hepatic neoplasms published until May 2021. The following electronic databases were explored: Medline through PubMed, Embase, The Cochrane Library, and MEDES; health technology assessment agencies, including the European Network for Health Technology Assessment (EUnetHTA), Network of Health Technology Assessment Agencies (REDETS), and the National Institute for Health and Care Excellence (NICE); and communications from international conferences, including the Cardiovascular and Interventional Radiological Society of Europe (CIRSE), European Conference on Interventional Oncology (ECIO), European Association of Nuclear Medicine (EANM), Society of Interventional Oncology (SIO), International Society for Pharmacoeconomics and Outcomes Research (ISPOR), European Congress of Radiology (ECR) and Society of Nuclear Medicine and Molecular Imaging (SNMMI).

### Inclusion and exclusion criteria

Studies that performed an economic evaluation of ^90^Y-TARE as a single treatment, as a combination treatment, or as part of a treatment sequence, regardless of the line of treatment, disease, or comparator, were considered. Studies that did not comply with the inclusion criteria were excluded. Economic evaluations that did not refer to ^90^Y-TARE as part of their development or evaluation were excluded. The inclusion and exclusion criteria were first applied to the titles and abstracts of the publications, and the full texts of the selected studies were reviewed.

### Data extraction

Two independent authors (NE and IO) executed the search strategy and independently screened all studies. Possible discrepancies after the review were resolved through discussion and consensus among the authors. Data was extracted using a standardized template (reviewed by NE and IO) and the parameters collected include author/s, year and country of publication, type of economic evaluation defined as full (cost-effectiveness-analysis [CEA], cost-utility analysis [CUA], and cost-minimization analysis [CMA]) and partial (cost-analysis [CA] and budget-impact-analysis [BIA]) economic evaluations, perspective, time horizon, type of model, evaluated comparative alternatives, patient characteristics, cost estimation, health outcomes, and cost-effectiveness results. Cost estimates were extracted as reported in the publication, converted to euros (€), and inflated to 2020 (€, 2020) using the reference exchange published by the European Central Bank. Inflation rates were derived from the Organisation for economic co-operation and development (OECD). To eliminate differences in the purchasing power across the different currencies and countries, a purchasing power parity factor (PPP) was performed to convert the costs to international dollars (US$ PPP) [18].

### Quality assessment

The methodological quality of the included studies was assessed using the Consolidated Health Economic Evaluation Reporting Standards (CHEERS) checklist [19]. CHEERS includes a 24-item checklist and assigns a score of 1 if the explicit parameters contemplated in the studies were met (“YES”) and a score of 0 if they were not (“NO”). The full (CEA, CUA, and CMA) economic evaluations were evaluated against a 24-item checklist, and the partial (CA and BIA) were evaluated against a 20-item-checklist. This difference was due to the 4 items (items 9, 10, 12, and 21) not being applicable to the study type. An internal classification criterion was developed to assess and categorize the quality of included studies as low (< 50%), medium (50% and 80%), and high (> 80%). The final included studies were independently reviewed by co-authors (NE and IO).

## Results

### Study selection

**Fig. 1:**
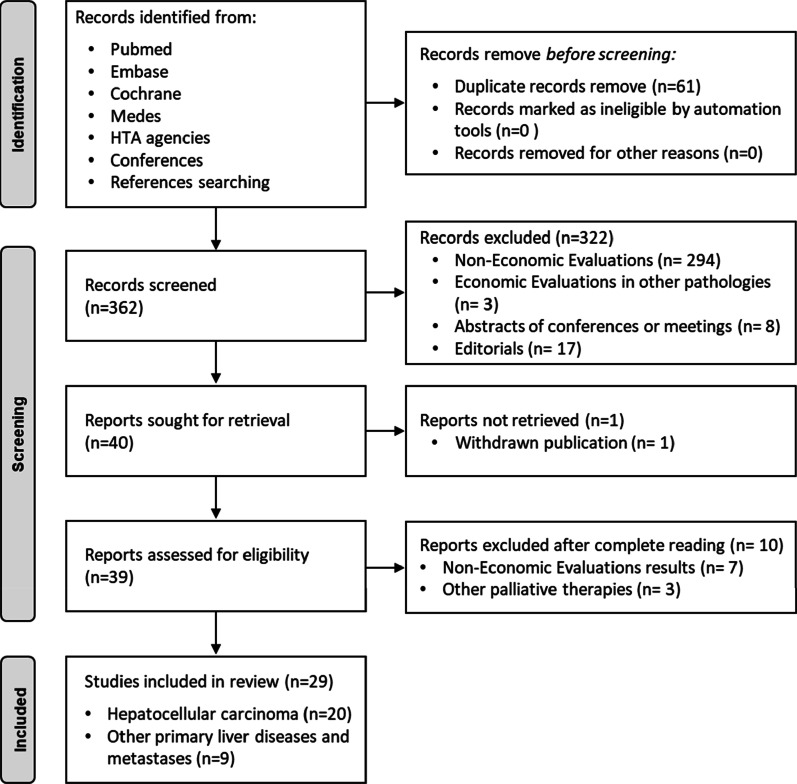
Bibliographic selection based on the PRISMA criteria

The database search identified 423 studies records, of which 394 were excluded as duplicates or did not meet the inclusion criteria. A total of 29 full-text studies were screened, of which nine studies were excluded due to: metastasis of colorectal cancer (n = 7), metastasis of neuroendocrine tumours of hepatic origin (n = 1), and intrahepatic cholangiocarcinoma (n = 1). Twenty studies met the eligibility criteria. A flow diagram of records founds, screened, selected, and full-text studies evaluated is shown in Fig. 1.

### Overview of the included studies

Eleven of the 20 studies (55%) were full economic evaluations [20–30] and nine studies (45%) were partial evaluations [31–39] (Table 1). Using the CHEERS checklist, the thirteen articles were of high quality (mean score of 94%), and seven abstracts/poster were of lower quality assessment (mean score of 56%), mainly because of the limited breadth of data.

**Table 1:** Quality assessment using the CHEERS statement checklist

	Section/item	Full economic evaluations											Partial economic evaluations								
		USA				Italy		United Kingdom					Canada	USA		Italy			United Kingdom		
		Rostambeigi 2014 [20]	Rostambeigi 2014 [21]	Parikh 2018 [27]	Marqueen 2021 [30]	Rognoni 2018 [23]	Rognoni 2017 [26]	Chaplin 2015 [24]	Palmer 2017 [25]	Walton 2020 [28]	Manas 2021 [22]	Muszbek 2020 [29]	Hubert 2016 [32]	Ray 2012 [34]	Ljuboja 2021 [35]	Colombo 2015 [31]	Lucà 2018 [36]	Rognoni 2018 [37]	Muszbek 2019 [33]	Muszbek 2021 [38]	Pollock 2020 [39]
		^a^	^b^	^b^	^a^	^a^	^a^	^b^	^b^	^a^	^a^	^a^	^b^	^a^	^a^	^a^	^a^	^a^	^b^	^b^	^a^
1	Title	1	1	1	1	1	1	1	1	1	1	1	1	1	1	1	1	1	1	1	1
2	Abstract	1	0	0	1	1	1	0	0	1	1	1	0	1	1	1	1	1	0	0	0
3	Background and objectives	1	1	1	1	1	1	1	1	1	1	1	1	1	1	1	1	1	1	1	1
4	Study population, objectives, and subgroups	1	1	1	1	1	1	1	1	1	1	1	1	1	1	1	1	1	1	1	1
5	Setting and location	1	1	1	1	1	1	1	1	1	1	1	1	1	1	1	1	1	1	1	1
6	Perspective	1	1	1	1	1	1	1	1	1	1	1	1	1	1	1	1	1	1	1	1
7	Comparators	1	1	1	1	1	1	1	1	1	1	1	1	1	1	1	1	1	0	1	1
8	Time horizon	1	1	1	1	1	1	0	0	1	1	1	1	1	1	1	1	1	1	0	1
9	Discount rate	0	0	0	1	1	1	0	0	1	1	1	NA	NA	NA	NA	NA	NA	NA	NA	NA
10	Selections of health outcomes	1	1	1	1	1	1	1	1	1	1	1	NA	NA	NA	NA	NA	NA	NA	NA	NA
11	Measurement of effectiveness	1	0	1	1	1	1	0	1	1	1	1	1	1	1	1	1	1	1	1	1
12	Measurement and valuation of preference-based outcomes	0	0	0	1	1	1	0	0	1	1	1	NA	NA	NA	NA	NA	NA	NA	NA	NA
13	Estimating resources and costs	1	1	1	1	1	1	1	1	1	1	1	1	1	1	1	1	1	1	1	1
14	Currency, price date, and conversion	0	0	0	1	1	1	0	0	1	1	1	1	1	1	1	0	1	1	1	1
15	Choice of model	0	0	0	1	1	1	0	0	1	1	1	1	1	1	1	1	1	1	1	1
16	Assumptions	1	0	0	1	1	1	0	0	1	1	1	0	1	0	0	0	1	0	0	0
17	Analytic methods	1	1	1	1	1	1	1	1	1	1	1	1	1	1	1	1	1	1	1	1
18	Study parameters	0	0	0	1	1	1	0	0	1	1	1	0	1	1	1	1	1	0	0	0
19	Incremental costs and outcomes	1	0	1	1	1	1	0	1	1	1	1	0	1	1	1	1	1	1	1	0
20	Characterizing uncertainty	1	0	1	1	1	1	1	1	1	1	1	0	1	1	0	0	1	0	0	0
21	Characterizing heterogeneity	0	0	0	1	1	1	0	0	1	1	1	NA	NA	NA	NA	NA	NA	NA	NA	NA
22	Discussion	1	1	0	1	1	1	0	0	1	1	1	0	1	1	1	1	1	1	0	0
23	Source of funding	0	0	0	1	1	1	1	1	1	1	1	0	0	1	1	1	1	0	0	0
24	Conflicts of interest	1	0	1	1	1	0	0	1	1	1	1	0	0	1	1	1	1	0	0	0
	Total	17	11	14	24	24	23	11	14	24	24	24	12	18	19	18	17	20	13	12	20
	% (n)	71%	46%	58%	100%	100%	96%	46%	58%	100%	100%	100%	60%	90%	95%	90%	85%	100%	65%	60%	100%

^a^Article

^b^Oral communications and abstracts

### Full economic evaluations (n = 11)

#### Characteristics of the included studies

Eleven publications were categorized as full economic evaluations (7 articles [20, 22, 23, 26, 28–30] and 4 congress communications [21, 24, 25, 27]). Seven were published from a European perspective [22–26, 28, 29] and four from the USA [20, 21, 27, 30]. The HCC population studied were mainly patients with HCC in the intermediate and advanced stages (8 of 11 publications: one BCLC-B [23], four BCLC-C [24, 25, 27, 30], and three grouped stages BCLC-B and BCLC-C [26, 28, 29]); one publication grouped early and intermediate stages [22], and two publications grouped all three stages (BCLC-A, B and C) [20, 21].

Regarding the type of microsphere evaluated, three publications did not specify the type of microsphere [21, 26, 27]; two studies referred to TheraSphere^®^ [22, 24], two studies referred to SIR-Spheres^®^ [25, 29], three studies referred to both types (TheraSphere^®^ and SIR-Spheres^®^) [20, 23, 30], and one study reported the use of three types of microspheres, including QuiremSpheres^®^ [28]. The main comparators were TACE [20–23] and sorafenib [24–30, 30], in addition to transarterial embolization (TAE) [22], TACE with doxorubicin-releasing particles (DEB-TACE) [22] and lenvatinib [28].

Regarding the pharmacoeconomic parameters, two of the eleven studies were CEA [20, 21], eight were ACU [22–24, 26–30], and one was a CMA [25]. Six of the eleven studies used a Markov modelling [22–24, 26, 27, 30], two studies utilized Monte-Carlo modelling [20, 21], two were survival-based models [28, 29], and one utilized decision trees modelling [28]. The cost minimisation study did not specify the type of model [25] used. The time horizon ranged from 5 years [20, 21, 30] to lifetime [23, 26, 27, 29]. The payer’s perspective predominated (10 of 11 publications), although one study focused on the social perspective [28]. The outcome measures included overall survival (OS), life month gained (LMG), life years gained (LYG), quality-adjusted life years (QALY), incremental cost-effectiveness ratios (ICERs), incremental cost-utility ratios (ICURs), willingness-to-pay (WTP), and incremental net monetary benefit (NMBs). The characteristics of the full economic evaluations are summarized in Table 2.

**Table 2:** Descriptive analysis of full economic evaluations for hepatocellular carcinoma

Author, year, publication type and country	Patient’s characteristics	Treatments		Analysis type/model	Perspective/time horizon	Cost	Outcomes
		Comparators	Microspheres				
**TARE versus TACE**							
Rostambeigi, 2014 [20] *Original article* USA	BCLC-A BCLC-B BCLC-C	TARE versus TACE	TheraSphere™ SIR-Spheres^®^	CEA/Monte Carlo	Payer/5 years	Direct cost (medical)	OS and incremental cost
Rostambeigi, 2014 [21] *Communication at congress* USA	BCLC-A BCLC-B BCLC-C	TARE versus TACE	ND	CEA/Monte Carlo	Payer/5 years	ND	OS, procedure- and complications costs, and incremental cost
Manas, 2021 [22] *Original article* United Kingdom	BCLC-A BCLC-B	TARE versus TACE, TAE o DEB-TACE	TheraSphere™	CUA/Markov	Payer/20 years	Direct cost (medical)	Downstaging^a^, LYG, QALY, ICER(£/LYG) y ICUR(£/QALY)
Rognoni, 2018 [23] *Original article* Italy	BCLC-B	TTS: TARE + TACE + sorafenib (on 47% of patients) TS: TARE + sorafenib	TheraSphere^™^ SIR-Spheres^®^	CUA/Markov	Payer/lifetime	Direct cost (medical)	Cost, QALY, ICUR (€/QALY), WTP a €50,000/QALY
**TARE versus TKIs**							
Chaplin, 2015 [24] *Communication at congress* United Kingdom	BCLC-C^b^	TARE versus sorafenib	TheraSphere^™^	CUA/Markov	Payer/10 years	ND	Cost, TTP, SG y ICUR (£/QALY),
Palmer, 2017 [25] *Communication at congress* United Kingdom	BCLC-C	TARE versus sorafenib	SIR-Spheres^®^	Cost-minimization analysis	Payer/ND	Direct cost (medical)	Cost (£), principals factors cost, QALY
Rognoni, 2017 [26] *Original article* Italy	BCLC-B BCLC-C	TARE versus sorafenib	ND	CUA/Markov	Payer/lifetime	Direct cost (medical)	Cost, QALY, ICUR (€/QALY), WTP a €38,500 (~ £30,000)/QALY
Parikh, 2018 [27] *Communication at congress* USA	BCLC-C^c^	TARE versus sorafenib	ND	CUA/Markov	Payer/lifetime	Direct cost (medical)	ICUR ($/QALY)
Walton, 2020 [28] *Systematic review an economic evaluation* United Kingdom	BCLC-B BCLC-C (*Child–Pugh A e ineligible a CTT)*	TARE versus TKIs	TheraSphere^™^ SIR-Spheres^®^ QuiremSpheres^®^	CUA/Partitioned survival model and decision tree	Payer and social/10 years	Direct and indirect cost	ICUR (£/QALY), incremental net monetary (NMB)
Muszbek, 2020–21 [29] *Original article* United Kingdom	BCLC-B^d^ BCLC-C^d^	TARE versus sorafenib	SIR-Spheres^®^	CUA/Partitioned survival model	Payer/lifetime	Direct cost (medical)	Cost, LYG, QALY, ICUR (£/QALY), WTP a £20.000, INB
Marqueen, 2021 [30] *Original article* USA	BCLC-C	TARE versus sorafenib	TheraSphere^™^ SIR-Spheres^®^	CUA/Markov	Payer/5 years	Direct cost (medical)	Cost, QALY, ICUR (€/QALY), WTP a $100,000/QALY o $200,000/QALY

*BCLC* Barcelona Clinic Liver Cancer classification, *CEA* cost-effectiveness analysis, *CTT* conventional transarterial therapy, *CUA* cost-utility analysis, *DEB-TACE* doxorubicin eluting bead transarterial chemoembolization, *HCC* hepatocellular carcinoma, *ICER* cost-effectiveness incremental ratio, *ICUR* incremental cost-utility ratio, *LYG LYG* life-years gained, *ND* no data, *OS* overall survival, *QALY* quality-adjusted life years, *TACE* transarterial chemoembolization, *TAE* transarterial embolization, *TARE* transarterial radioembolization, *TKI* tyrosine kinase inhibitors, *TTP* time to progression, *TTS sequency* TARE, TACE and optional sorafenib (sorafenib was administered on 47% of patients), *WTP* willingness-to-pay

^a^Downstaging: decrease in tumour burden that allows patients to be rescued for treatments such as liver transplantation

^b^Assumed clinical characteristics of two separate RCTs: TheraSphere (Salem et al. 2011) and sorafenib (Phase III SHARP RCT-Llovet et al. 2018)

^c^Patients with unresectable HCC and Child–Pugh class A cirrhosis

^d^BCLC-B o BCLC-C (not appropriate to TACE): HCC with low tumour burden (≤ 25%) and good liver function (albumin–bilirubin [ALBI] grade 1)

##### TARE versus TACE

TACE therapy was one of the comparators considered in four of the eleven studies [20–23]); two studies [20, 21] compared TARE with TACE, a third study [22] included TACE and two other comparators (TAE and DEB-TACE), and lastly publication reported TACE as part of a sequence of therapies (TARE, TACE and possibly sorafenib [TTS sequence] versus TARE plus sorafenib [TS sequence]) [23]. The stages of the evaluated patients were heterogeneous; early [20–22], intermediate [20–23], and advanced [20, 21] disease.

##### TARE versus TKI

Seven studies [24–30] used systemic therapy as a comparator; 6 studies [24–27, 29, 30] reported only sorafenib as a comparator, and one study [28] included lenvatinib. Additionally, these seven studies evaluated patients with the intermediate-advanced disease.

#### Results of the full economic evaluations

The costs and health outcomes reported in the eleven studies were heterogeneous (Table 3).

**Table 3:** Results of full economic evaluations for hepatocellular carcinoma

Author, year publication (year cost)	Stage	Comparators	Costs		Outcome’s health		Ratio cost/outcome’s health			
			Original cost	Adjusted to $US PPP [18]	LYG	QALY	ICER €/LYG	ICUR €/QALY	ICER $US PPP/LYG	ICUR $US PPP/QALY
**TARE versus TACE**										
Rostambeigi, 2014 [20] (2013)^﻿a^			***Monthly***^**b**^		***OS months***					
	BCLC-A	TACE	$ 2094	2347	39.5	ND	TACE versus	ND	TACE versus	ND
		TARE (I)	$ 1770	1311	29.7	ND	$33/LMG	ND	37/ LMG	ND
			Δ − $ 324	Δ − 363	Δ 9.8		[$ 396 LYG]*		[444/LYG]*	
		TARE (II)	$ 2688	3013	29.7	ND	$61/LMG	ND	68/LMG	ND
			Δ $ 594	Δ 666	Δ 9.8		[− $ 732 LYG]*		[− 820/LYG]*	
	BCLC-B	TACE	$ 2326	2607	22.9	ND	TACE versus		TACE versus	
		TARE (I)	$ 2789	3126	16.0	ND	$67/LMG	ND	75/LMG	ND
			Δ $ 463	519	Δ 6.9		[− $ 804 LYG]*		[− 901/LYG]*	
		TARE (II)	$ 4240	4753	16.0	ND	$277/LMG	ND	310/LMG	ND
			Δ $1914	2145	Δ 6.9		[− $3324 LYG]*		[− 3726/LYG]*	
	BCLC-C	TACE	$ 2679	3003	13.3	ND	TACE versus		TACE versus	
		TARE (I)	$2652	2973	17.1	ND	$7/LMG	ND	8/LMG	ND
			Δ − $27	Δ − 30	Δ 3.8		[Dominant]*		[Dominant]*	
		TARE (II)	$4031	4518	17.1	ND	$356/LMG	ND	399/LMG	ND
			Δ $1352	Δ 1515	Δ 3.8		[$ 4272 LYG]*		[− 4788/LYG]*	
Rostambeigi, 2014 [21] (2013)^a^					***OS months***					
	BCLC-A, BCLC-B, and BCLC-C	TACE	$ 17,000	19,055	BCLC-A: 37 BCLC-B: 22 BCLC-C: 12	ND	ND	ND	ND	ND
		TARE	$ 49,000	54,924	BCLC-A: 32 BCLC-B: 18 BCLC-C: 19	ND	ND	ND	ND	ND
	BCLC-C	TARE-TACE	Δ $ 500	Δ 560		ND	ND	ND	ND	ND
Manas, 2021 [22]^c^ (2020)	BCLC-A, BCLC-B	TARE (T™)	£ 49,583	49,921	3.05	2.24	TARE versus	TARE versus	TARE versus	TARE versus
		TACE	£ 37,038	37,291	2.14	1.57	£ 12,808	£ 17,279	12,291	17,397
		DEB-TACE	£ 33,206	33,432	2.14	1.57	£ 17,059	£ 23,020	17,175	23,177
		TAE	£ 37,015	37,267	2.14	1.57	£ 12,833	£ 17,300	12,921	17,418
					Δ 0.91	Δ 0.67	WTP (£20.000/QALY): 15.9% (TARE vs. DEB-TACE) to 76.8% (TARE vs. TACE) WTP (£30.000/QALY): 88.6% (TARE vs. DEB-TACE) to 98.7% (TARE vs. TAE)			
Rognoni, 2018 [23] (2016)	BCLC-B	TTS (47% sorafenib)	€ 36,509	37,137	3.494	1.385	–	TTS Dominant		
		TS	€ 42,812	43,591	2.361	0.937				
			Δ − € 6303	Δ − 6418	Δ − 1.133	Δ 0.448	TTS WTP (€50,000/QALY): 83%			
**TARE versus TKI**										
Chaplin, 2015 [24] (2015)^a^	BCLC-C	TARE (T™)	£ 21,441	22,763	ND	1.12	ND	TARE Dominant	ND	TARE Dominant
		Sorafenib	£ 34,050	36,150	ND	0.85	ND			
			Δ − £ 12,609	Δ − 13,387	ND	Δ 0.27	ND			
					TARE versus sorafenib TTP (months): 6.2 versus 4.9 OS (months): 13.8 versus 9.7					
Palmer, 2017 [25] (2017)	BCLC-C	TARE (S^®^)	£ 8909 in favour of TARE	9374 favour of TARE	ND	Δ 0.0079 in favour of TARE	ND	TARE cost-effective	ND	TARE cost-effective
		Sorafenib								
			Cost drivers: workup and administrations for TARE and duration of treatment for sorafenib							
Rognoni, 2017 [26] (2015)	BCLC-B	TARE	€ 31,071	31,644	2.531	1.178	TARE versus	TARE versus	TARE versus	TARE versus
		Sorafenib	€ 29,289	29,829	1.575	0.638	1865	3302	1899	3363
			Δ € 1782	Δ 1815	Δ 0.956	Δ 0.540	WTP (€38500/QALY): 99.2%			
	BCLC-C	TARE	€ 21,961	22,366	1.445	0.639	ND	TARE Dominant	ND	TARE Dominant
		Sorafenib	€ 30,750	31,317	1.306	0.568				
			Δ − € 8788	Δ − 8950	Δ 0.139	Δ 0.071	WTP (€38.500/QALY): 98.2%			
Parikh, 2018 [27] (2018)^a^	BCLC-C	**Pooled data**						Sorafenib versus		Sorafenib versus
		TARE	$ 61,897	65,295	ND	0.81	ND	$ 19,534	ND	20,606
		Sorafenib	$ 63,313	66,789	ND	0.88				
			Δ − $ 1416	Δ − 1494	ND	Δ − 0.07				
		**CT SARAH**						Sorafenib versus		Sorafenib versus
		TARE	$ 64,805	68,363	ND	0.78		TARE versus		TARE versus
		Sorafenib	$ 63,216	66,687	ND	0.87	ND	Sorafenib Dominant	ND	Sorafenib Dominant
			Δ $ 1589	Δ 1676	ND	Δ − 0.09				
		**CT SIRveNIB**						Sorafenib versus		Sorafenib versus
		TARE	$ 57,473	60,628	ND	0.84	ND	$ 107,927	ND	113,852
		Sorafenib	$ 63,447	66,930	ND	0.90				
			Δ − $ 5974	Δ − 6302	ND	Δ − 0.06				
Walton, 2020 [28] (2017/2018)	BCLC-B and BCLC-C	**Deterministic**								
		TARE (T™)	£ 29,888	30,922	1.110	0.764	*NMB (£)*	TARE (T™) versus	*NMB (£)*	TARE (T™) versus
		TARE (S^®^)	£ 30,107	31,148	1.110	0.764	*−* *218*	+ Costly	*226*	+ Costly
		TARE (Q^®^)	£ 36,503	37,766	1.110	0.764	*−* *6614*	+ Costly	*−* *6843*	+ Costly
		Lenvatinib	£ 30,005	31,043	1.243	0.841	*97*	28,728	*100*	29,722
		Sorafenib	£ 32,082	33,192	1.183	0.805	*1090*	2911	*1128*	3012
		**Probabilistic**								
		TARE (T™)	£ 30,014	31,052	1.111	0.765	*NMB (£)*	TARE (T™) versus	*NMB (£)*	TARE (T™) versus
		TARE (S^®^)	£ 30,196	31,240	1.111	0.765	*−* *2154*	Dominated	*−* 2229	Dominated
		TARE (Q^®^)	£ 36,613	37,879	1.111	0.765	*−* *2323*	Dominated	*−* 2403	Dominated
		Lenvatinib	£ 29,658	30,684	1.244	0.841	*−* *2306*	174,320	*−* 2386	180,349
		Sorafenib	£ 32,444	33,566	1.202	0.825	*−* *8741*	Dominated	*−* 9043	Dominated
Muszbek, 2020–21 [29]^d^ (2018/2019)	BCLC-B and BCLC-C	TARE (S^®^)	£ 29,530	30,085	2.637	1.982		TARE Dominant		TARE Dominant
		Sorafenib	£ 30,957	31,539	1.890	1.381	ND	*−* £ 2374	ND	−2719
			Δ − £ 1427	Δ − 1454	Δ 0.748	Δ 0.601	TARE (S^®^) WTP (£ 20,000): 95%. INB (£) at threshold of £20,000: £ 13,443			
Marqueen, 2021 [30] (2016/2017)	BCLC-C	**Pooled data**								
		Sorafenib	$ 78,859	84,868		0.88		Sorafenib versus		Sorafenib versus
		TARE	$ 58,397	62,847		0.87	ND	$ 1,280,224	ND	1,377,777
			Δ $20,462	Δ 22,061		Δ 0.02	Sorafenib WTP ($200,000/QALY): 1%			
		**CT SARAH**								
		Sorafenib	$ 72,899	78,454		0.83		Sorafenib versus		Sorafenib versus
		TARE	$ 66,800	71,890		0.84	ND	TARE dominant	ND	TARE dominant
			Δ $ 6099	Δ 6564		Δ − 0.01				
		**CT SIRveNIB**								
		Sorafenib	$ 89,806	96,649		0.91		Sorafenib versus		Sorafenib versus
		TARE	$ 46,151	49,668		0.86	ND	$ 753,412	ND	810,822
			Δ $43,655	Δ 46,982		Δ 0.06				

*BC* base case, *BCLC* Barcelona Clinic Liver Cancer classification, *CT* clinical trial, *DEB-TACE* doxorubicin eluting bead transarterial chemoembolization, *HCC* hepatocellular carcinoma, *CI* confidence interval, *ICER* cost-effectiveness incremental ratio, *ICUR* incremental cost-utility ratio, *INB* incremental net benefit, *LYG* life years gained, *LMG* life moth gained, *ND* no data, *NMB* net monetary benefit, *OS* overall survival, *QALY* quality-adjusted life years, *TACE* transarterial chemoembolization, *TAE* transarterial embolization, *TARE* transarterial radioembolization, *TARE (I)* unilobar, *TARE (II)* bilobar, *TARE (S*^*®*^*)* transarterial radioembolization with SIR-Spheres^®^, *TARE (T™)* transarterial radioembolization with TheraSphere™, *TARE (Q*^*®*^*)* transarterial radioembolization with QuiremSpheres^®^, *TKI* tyrosine kinase inhibitors, *TTP* time to progression, *TTS sequency* TARE, TACE and optional sorafenib (sorafenib was administered on 47% of patients), *WTP* willingness-to-pay

*Determined by calculations assuming a year has 12 months

^a^Year of unspecified cost, estimated from the proposed cost reference sources

^b^The procedure is repeated every 10 months until 5 years

^**c**^Number of patients downstaged (out of 1000 patients): 842 TheraSphere™ and 452 TACE, DEB-TACE and TAE

^d^TARE allows downstaging for subsequent treatment with curative intent: 13.5% TARE versus 2.1% sorafenib (base case considering SARAH study data), and 5.1 TARE versus 1.4% sorafenib in the ITT population

##### TARE versus TACE

Four studies reported higher costs (TARE versus TACE) [20–22], and this finding was independent of the patient's BCLC-A, B, or C in three studies. The fourth publication presented a higher cost in TS sequence therapy than TTS sequence (47% of patients with sorafenib) in patients with the intermediate disease [23].

In one study, the health outcomes reported for patients in the intermediate stage showed a benefit of TARE over TACE in terms of LYG and QALY [22]. The study evaluated sequences of therapies, TTS (with optional sorafenib), and showed a greater incremental benefit than TS for LYG and QALYs [23]. Two studies [20, 21]) reported the benefits for TARE in the advanced stage (BCLC-C), with lower benefits compared to TACE in the early and intermediate stages.

The ICERs of TARE versus TACE presented monthly (LMG) [20] and annual costs (LYG) [22]. Additionally, two studies [22, 23] presented ICUR results (€/QALY), and one study did not present any ratios [21]. For the early and intermediate stages of the disease, one study (Manas et al. [22]) presented an ICER of £ 12,833/LYG (£, 2020) (12,291 $US PPP/LYG) and established the ICUR of TARE versus TACE at £ 17,279/QALY (£, 2020) (17,397 $US PPP/QALY), with a 76.5% probability of being profitable considering a cost-effectiveness threshold of £ 20,000/QALY (£, 2020). In the intermediate stage, one study evaluated two treatment sequences and reported that TTS (with sorafenib in 47% of patients), including TARE, was the dominant strategy (i.e., it offered greater effectiveness with lower associated cost). When compared to TS, an 83% probability of being efficient based on a threshold of € 50,000/QALY was estimated [23]. In the advanced stage, TARE was superior to TACE (ICER 8 $US PPP/LMG) when the intervention was evaluated in one lobe and obtained an ICER of $ 356/LMG ($, 2013) (399 $US PPP/LMG) when the two-lobe intervention was evaluated [20]. TARE was inferior (with lower effectiveness and higher associated cost) when used in the early and intermediate stages [20]. The second publication by Rostambeigi et al. [21] did not detail the calculation of ICERs.

##### TARE versus TKI

Six [24–26, 28–30] of the seven studies compared TARE with sorafenib in patients with intermediate-advanced stage and reported lower costs for TARE (differences between 1454 to 46,982 $US PPP). However, Parikh et al. [27] evaluated a similar group of patients and reported conflicting cost results, a difference attributable to the source of the clinical trial efficacy parameters.

The benefits for health outcomes were greater for TARE [24–26, 29] than sorafenib in four of the seven studies (maximum QALY gained was 0.540 in BCLC-B, 0.27 in BCLC-C, and 0.601 in both stages); two studies [27, 28] showed greater health benefits for sorafenib (maximum QALY gained was 0.09), and one study [30] reported differing results depending on the source of clinical efficacy.

For patients with advanced-stage, TARE therapy was considered superior to sorafenib in five [24–26, 29, 30] of the seven studies when the SARAH RCT clinical parameters were used [7] as the source of clinical efficacy. The remaining two studies [27, 28] reported sorafenib was superior to TARE in patients with intermediate-advanced stage.

#### Study quality reporting assessment

Included studies categorized as full economic evaluations were appraised for their quality: six of the eleven studies (55%) [22, 23, 26, 28–30] had a high score when evaluated with the 24-item checklist (mean compliance: = 99%). Approximately, 27% (3 of 11) and 18% (2 of 11) of the studies had a moderate score (mean compliance: 66%) [20, 25, 27] and a low score (mean compliance of 46%) [21, 24], respectively.

### Partial economic evaluations (n = 9)

#### Characteristics of the included studies

Nine publications were partial evaluations (6 articles [31, 34–37, 39] and 3 congress communications [32, 33, 38]). Six publications were from the European perspective [31, 33, 36–39]), two from the United States [34, 35], and one from the Canadian perspective [32]. The HCC population included patients with intermediate and advanced stages in seven of the nine studies [31–33, 36–39]; five studies [31, 32, 36, 37, 39] reported the inclusion of patients as BCLC-B or BCLC-C, and two studies defined the intermediate or advanced stage as unresectable HCC (Muszbek et al.) [33, 38]. Of the two remaining studies, one (Ray et al.) [34] described HCC in a way that can be assumed to correspond to an early BCLC-A stage (male patient 65 years old with unresectable solitary HCC of 3 cm isolated in 1 lobe, not suitable for transplantation), and the second study (Ljuboja et al.) [35] did not define the population.

Three of the nine studies evaluated SIR-Spheres^®^ [31, 35, 39], one included TheraSphere^®^ [32], three considered both TheraSphere^®^ and SIR-Spheres^®^ [36–38], and two did not specify the type of microsphere evaluated. The comparators were TACE [31, 32, 34, 35, 38], ablative therapy [34, 35] and systemic therapies (sorafenib [31, 33, 36, 37, 39] and lenvatinib [39]).

Regarding the time horizon, six studies were CA [31, 33–36, 38] and reported time horizons ranging from 1 month to 2 years. The remaining three studies were BIA [32, 37, 39] and reported time horizons ranging from 3 years to a lifetime horizon. The payer’s perspective was most frequently used (100%); with the exception of one study that considered the social perspective [38]. The HCC stages of the study population, the comparators, and the outcome measures considered in the partial economic evaluations are highlighted in Table 4.

**Table 4:** Descriptive analysis of partial economic evaluations for hepatocellular carcinoma

Author, year, publication type and country	Patient’s characteristics	Treatments	Microspheres	Analyses type/characteristics, source, and costs	Perspective/ time horizon	Outcomes
**TARE versus TACE and ablative therapy**						
Ray, 2012 [34] *Original article* USA	BCLC-A^a^	TARE versus TACE versus RFA	ND	CA/ Multiple scenarios for Medicare using a decision tree and Monte Carlo model Direct healthcare cost: Medicare reimbursement for hospital and repeat procedures comes from the literature	Payer/ 2 years	Estimated cost of each procedure Repetition rate to consider a strategy as optimal
Ljuboja, 2021 [35] *Original article* USA	ND	TARE versus TACE versus ablative therapy	SIR-Spheres^®^	CA/TDABC (retrospective and prospective) carried out in a tertiary care hospital Direct health costs: In-hospital costs (from admission to discharge) of the treatments evaluated	Payer/1 year	Estimated cost of each procedure (estimate of 4 patients per alternative evaluated) Cost drivers
**TARE versus TACE and/or TKI**						
Colombo, 2015 [31] *Original article* Italy	BCLC-B and BCLC-C	TARE versus TACE versus Sorafenib	SIR-Spheres^®^	CA/Retrospective in 4 centres. Data from 137 patients [BCLC-B (n = 80) and BCLC-C (n = 57)] out of a total of 285 Direct healthcare costs: Cost of treatments (TARE, TACE and sorafenib) and associated drugs, diagnostic and laboratory tests, administration (consumables and professionals) and monitoring (visits)	Payer/ 1 year	Estimated cost of each procedure Average number of treatments per year
Muszbek, 2019 [33] *Communication at congress* United Kingdom	BCLC-B^b^	TARE versus TACE	TheraSphere™ SIR-Spheres^®^	CA/Multiple scenarios of resource consumption (retrospective and expert) and costs (reference costs or microcosting) Direct health costs: Cost of treatments, administration, management of AE and hospitalisation costs	Payer/ ND	Estimated cost range for each alternative Cost drivers
Hubert, 2016 [32] *Communication at congress* Canada	BCLC-B	TARE versus TACE^e^	TheraSphere™	BIA/Epidemiological of a hospital Direct healthcare costs: Cost of treatments (pharmacological and devices), administration (key cost drivers) and management of AE	Payer/ 3 years	Annual (reimbursement) cost per alternative for a hospital treating 200 HCC patients annually
	BCLC-C^c^	TARE versus sorafenib				
**TARE versus TKI**						
Lucà, 2017 [36] *Original article* Italy	BCLC-B BCLC-C	TARE versus sorafenib	TheraSphere™ SIR-Spheres^®^	CA/Retrospective observational study (one centre), comparing a subgroup of sorafenib (SOR3)^d^ with the TARE group Direct healthcare costs: Cost of treatments (drug and devices), administration, monitoring and hospitalisation costs	Payer/272 days	Estimated cost of each procedure OS rates
Muszbek, 2019 [38] *Communication at congress* United Kingdom	BCLC-C^b^	TARE versus sorafenib	ND	CA/Costs by health status obtained from literature, registers, and surveys (5 experts) Direct health costs (historical and current): administration, monitoring and hospitalisation costs Social care	Payer y social/ 1 month	Comparative cost of resources by state of health between 2007 and 2015
Rognoni, 2018 [37] *Original article* Italy	BCLC-B (Post-TACE) BCLC-C^c^	TARE versus sorafenib	TheraSphere™ SIR-Spheres^®^	BIA/Markov Source: Three Italians oncology centres Direct healthcare costs: Cost of treatments (pharmacological and devices), administration, monitoring, hospitalisation costs and AE management and second-line treatments	Payer/5 years and lifetime	Estimated cost of each procedure Economic impact No. of deaths avoided No. of hospitalisations
Pollock, 2020 [39] *Original article* United Kingdom	BCLC-B *(not eligible to TACE)* BCLC-C *(eligible)*	TARE versus TKIs [95% sorafenib/ lenvatinib 5%]	SIR-Spheres^®^	BIA/Markov Source: CT SARAH	Payer/3 years	Economic impact in Spain, France, Italy and United Kingdom

*AE* adverse events, *BIA* budget impact analysis, *CA* cost analysis, *CT* clinical trial, *ND* no data, *RFA* radiofrequency ablation, *SOR* subgroup of patients with sorafenib, *TACE* transarterial chemoembolization, *TAE* transarterial embolization, *TARE* transarterial radioembolization, *TKI* tyrosine kinase inhibitors, *TDABC* time-drive activity-based costing

^a^BCLC classification not specified, stage interpreted according to patient type characteristics (3 cm isolated HCC in one lobe)

^b^Unspecified BCLC classification, stage interpreted according to pathology and comparator characteristics (TACE-eligible unresectable HCC). BCLC-C stage with and without portal vein thrombosis

^c^Advanced with tumour macrovascular invasion without extrahepatic spread and good liver function

^d^Patient flow: total patients treated with sorafenib (SOR) were divided into two groups according to treatment duration (SOR1 ≤ 2 months, SOR2 > 2 months). SOR2 patients who met criteria for TARE treatment (unilobar HCC, no metastases) were reassigned to SOR3 (24 patients: 54% BCLC-B, 46% BCLC-C)

^e^Consider conventional TACE or DEB-TACE

##### TARE versus TACE

Treatment with TACE was considered as a comparator in five [31–35] of the nine studies. Four of five studies reported the stages of HCC (early [34], intermediate, and/or advanced stages [31–33]). In studies of intermediate-stage HCC, one study compared only TACE versus TARE [33], two studies [31, 32] included sorafenib in addition to TACE, and two studies [34, 35] reported including radiofrequency ablation (RFA).

##### TARE versus TKI

Four studies [36–39] used systemic therapy as a comparator: three [36–38] reported sorafenib as a comparator, while one [39] publication also included lenvatinib in the assessment. All four studies considered patients in the intermediate-advanced stage.

#### Results of the partial economic evaluations

The costs and health outcomes were heterogeneous, mainly due to the type of economic evaluation performed and the grouping of patients with the different stages of the disease. Aggregated data for intermediate and advanced stages (BCLC-B combined with BCLC-C) were reported in five studies [31, 32, 36, 37, 39]. Data differentiated by HCC stages was reported in three studies (BCLC-A [34], BCLC-B [33], and BCLC-C [38]), and one publication [35] did not report the stage of disease (Table 5).

**Table 5:** Results of partial economic evaluations for hepatocellular carcinoma

Author, year publication (year cost)	Stage	Comparators	Costs								Resource consumption and health outcomes
			Original cost				Adjusted to $US PPP [18]				
**TARE versus TACE versus ablative therapy**											
Ray, 2012 [34] (2010﻿)	BCLC-A^a^		**Decision tree**		**Monte Carlo**		**Decision tree**		**Monte Carlo**		Threshold of repetitions to considered TARE an optimal strategy: – TARE repetition rate: 1–10% – TACE repetition rate: 82–77% TARE would be an optimal strategy versus TACE in 33.4 to 36.4% of cases
		TARE	$ 35,618		$ 35,629 ± 9930		42,368		42,381 ± 11,812		
		TACE	$ 30,143		$ 30,107 ± 19,109		35,855		35,812 ± 22,730		
		RFA	$ 9361		$ 9362 ± 2555		11,135		11,136 ± 3309		
Ljuboja, 2021[35] (2020)^b^	ND		**Total cost/patient**	**Personal**	**Equipment**	**Consumables**	**Total cost/patient**	**Personal**	**Equipment**	**Consumables**	Consumables reported for the highest cost in all three procedures, with a single consumable accounting for more than 30% of the total cost of each procedure
		TARE	$20,818 (100%)	$ 1656 (8%)	$ 371 (2%)	$ 18,791 (90%)	21,074	1676	376	19,022	
		TACE	$ 5089 (100%)	$ 1947 (38%)	$ 212 (4%)	$ 2930 (58%)	5152	1971	215	2966	
		Ablation	$ 3744 (100%)	$ 1114 (30%)	$ 205 (5%)	$ 2425 (65%)	3790	3837	208	2455	
**TARE versus TACE and/or TKI**											
Colombo, 2015 [31] (2014)	BCLC-B BCLC-C		**Annual cost/patient**		**Monthly cost/patient**		**Annual cost/patient**		**Monthly cost/patient**		Average number of treatments per year:
		TARE	26,106 €		17,404 €		26,629		17,753		TARE 1.50
		TACE	13,418 €		5304 €		13,687		5410		TACE 2.53
		Sorafenib	12,215 €		2009 €		12,460		2,049		Sorafenib 6.08
Muszbek, 2019 [33] (2018/2019)	BCLC-B^b^		**Annual cost/patient**				**Annual cost/patient**				The main cost driver is the number of TARE procedures per patient: TARE (glass): 1.08–1.20 TARE (resin): 1.20–1.58
		TARE (T™)	£ 12,026–£ 21,425				12,442–22,166				
		TARE (S^®^)	£ 11,185–£ 15,636				11,572–16,177				
		TACE	£ 9257–£ 14,167				9577–14,657				
Hubert, 2016 [32] (2016)^b^	BCLC-B BCLC-C	TARE, TACE and sorafenib	BIA HCC patients (n = 200 annual)^c^. TARE saved:				BIA HCC patients (n = 200 annual). TARE saved:				Costs at 3rd year (n = 200 patients) were device acquisition ($ 207,000 [227,526 $US PPP]); administration cost savings of $ 281,000 (308,864 $US PPP) and AE management savings of $ 1000 (1099 $US PPP)
			Year 1: $ 37,000				Year 1: 40,699				
			Year 2: $ 55,000				Year 2: 64,454				
			Year 3: $ 75,000				Year 3: 82,437				
			TARE was associated with cost savings and reduced use of hospital resources								
**TARE versus TKI**											
Lucà, 2017 [36] (2017)^b^	BCLC-B BCLC-C		**Total cost per patient**				**Total cost per patient**				At 2 years, the survival rate of TARE versus sorafenib SOR3 was significantly higher (p = 0.012). There was no significant difference in OS in the Kaplan–Meier analysis of SOR3 and TARE (*p* = 0.446)
		TARE	€ 17,761				18,096				
		Sorafenib (SOR3)	€ 27,992				28,520				
			TARE cost was significantly lower than sorafenib (*p* = 0.028). Limitations: small number of patients (n = 24) and the lack of randomisation in treatment type assignment								
Muszbek, 2019 [38] (2018/2019)	BCLC-C^d^		**Health status cost per month**				**Health status cost per month**				Costs 2007/2015 versus costs 2018/2019: Monthly cost is lower in the pre-progression and post-progression states (by 55% and 80%, respectively), due to reduced hospitalizations and social care
			**Pre**	**Progression**	**Post**		**Pre**		**Progression**	**Post**	
		TARE	£ 246	£208	£499		251		212	508	
		TKI	£ 287	£208	£287		292		212	292	
			Cost drivers in pre- and post-progression 2018/2019: diagnostic procedures (53%) and medical consultations (45%) 2007/2015: hospitalisations (41%) and social care (42%)								
Rognoni, 2018 [37] (2018)			**5 years**		**Lifetime**		**5 years**		**Lifetime**		Considering TARE/sorafenib utilisation rates of 30%/70% (year 1), 40%/60% (year 3) and 50%/50% (year 5–10), it was estimated: – Nº. deaths avoided: 2 in 5 years and 14 in 10 years – Nº of hospitalizations avoided due to hepatic decompensation: 32 in 5 years
	BCLC-B	TARE	€ 33,040		€ 28,003		33,393		28,302		
		Sorafenib	€ 29,935		€ 29,716		30,255		30,034		
	BCLC-C	TARE	€ 22,526		€ 21,456		22,767		21,685		
		Sorafenib	€ 31,526		€ 31,430		31,863		31,766		
	BCLC-B, BCLC-C	**BIA considering increased use of TARE (stage BCLC-B and C):**					**BIA considering increased use of TARE:**				
		Year 0 (TARE 20%, SOR 80%):			€ 30,139,457		Year 0		30,461,565		
		Year 1 (TARE 30%, SOR 70%):			€ 29,633,336		Year 1		29,950,035		
		Year 2 (TARE 30%, SOR 70%):			€ 29,239,463		Year 2		29,551,953		
		Year 3 (TARE 40%, SOR 60%):			€ 28,685,595		Year 3		28,992,165		
		Year 4 (TARE 40%, SOR 60%):			€ 28,311,921		Year 4		28,614,498		
		Year 5 (TARE 50%, SOR 50%):			€ 27,793,820		Year 5		28,090,860		
Pollock, 2020 [39] (2018)	BCLC-B, BCLC-C	**BIA at 3 years**	**France** (n = 699)	**Italy** (n = 629)	**Spain** (n = 497)	**UK** (n = 465)	**France** (n = 699)	**Italy** (n = 629)	**Spain** (n = 497)	**UK** (n = 465)	The highest resource consumption was: – Scenario without TARE: pharmacological cost – Scenario with TARE: pharmacological cost, work-up and procedure cost with TARE In Spain, higher total costs mainly derived from the management of AE grade 3 and 4 Proportion of HCC patients who ultimately receive treatment with curative intent for TARE was 4.6% and for TKIs was 1.4%
		With TARE	€ 23,234,726	€ 21,323,136	€ 18,905,157	£ 15,746,274	23,816,048	21,551,022	21,597,385	16,290,893	
		Without TARE	€ 26,314,378	€ 22,531,440	€ 25,172,537	£ 17,054,914	26,972,751	22,772,239	25,496,295	17,644,796	
		Cost savings (with vs. without TARE)	11.7%	5.4%	26.5%	7.7%					

*AE* adverse events, *BCLC* Barcelona Clinic Liver Cancer classification, *BIA* budget impact analysis, *HCC* hepatocellular carcinoma, *IHS* Italian health system, *ND* no data, *OS* overall survival, *RFA* radiofrequency ablation, *SOR* sorafenib, *SOR3* subgroup of patients with sorafenib, *TACE* transarterial chemoembolization, *TARE* transarterial radioembolization, *TKI* tyrosine kinase inhibitors

^a^BCLC classification not specified, stage interpreted according to patient type characteristics (3 cm isolated HCC in one lobe)

^b^Cost year not specified, estimated from the proposed cost reference sources

^c^The BIA considering 200 annual HCC patients (66% were treatment-eligible patients, of which 8, 13 and 17 patients were treated with TARE in years 1, 2 and 3, respectively)

^d^Unspecified BCLC classification, stage interpreted according to pathology and comparator characteristics (TACE-eligible unresectable HCC)

##### TARE versus TACE

Four CAs [31, 33–35] and one BIA [32] compared TARE versus TACE. The CA studies mostly indicated higher treatment costs (range: 11,572–42,368 $US-PPP) with TARE than with TACE (range: 9577–35,855 $US PPP) treatments [31, 33–35], ablative therapy (range: 3790–11,135 $US PPP) [34, 35] or sorafenib (12,460 $US PPP) [31]. However, one study (Muszbek et al.) [33] reported similar costs for TARE and TACE regardless of whether the costs were obtained from the official source (the NHS) or via a micro-costing approach [40]. Furthermore, Colombo et al. [31] highlighted the omission of the costs of unplanned hospitalization and adverse events (AEs) from their assessment. However, Ray et al. [34] established that in the early stage (based on a hypothetical cohort of patients older than 65 years) TARE had lower costs than TACE in more than one-third of the simulations of the evaluated scenarios. The BIA [32] study found cost savings with TARE during 3 consecutive years (savings of 40,699; 64,454, and 82,437 $US PPP at years 1, 2, and 3, respectively) of evaluation in a simulated population of 200 patients in a Canadian hospital.

No health outcomes were reported in the five studies that compared TARE with TACE. However, Colombo et al. [31] evaluated the treatment patterns in four centres in Italy and found TACE as the treatment of choice for intermediate HCC and sorafenib as the most commonly used first-line treatment for advanced HCC.

##### TARE versus TKI

The cost comparisons of TARE versus TKI (2 CA [36, 38] and 2 BIA [37, 39]) reported dissimilar results for TARE in patients with intermediate and/or advanced-stage disease. The CA by Lucà et al. [36] reported significantly lower cost for TARE (18,096 $US PPP) than sorafenib subgroup (28,520 $US PPP). Besides, the CA by Muszbek et al. [38] identified significant changes in the clinical practices for the management of advanced HCC patients, showing a 54 to 79% decrease in monthly costs compared to previous surveys. The BIA published by Rognoni et al. [37] from the Italian Health perspective was estimated to save € 7 million with the progressive increase in the use of TARE (from 20 to 50%) instead of sorafenib over 5 years. The second BIA (Pollock et al.) [39] evaluated TARE versus without TARE in four European countries (Spain, France, Italy, and the United Kingdom) and reported the use of TARE in Spain would generate a cost savings of 26.5% over a 3-year period.

Within the type of resources used, the pharmacological cost, the work-up, the number of procedures and the management of AEs were identified as cost drivers for TARE and TKIs. Only three [36, 37, 39] of the four studies provided health outcomes in the survival rates [36], the number of events (deaths or hospitalizations) avoided [37], incremental LYG [39], and the proportion of patients receiving treatment with curative intent [39]. The CA by Lucà et al. [36] estimated that TARE had significantly higher medium-term survival rates than sorafenib (TARE 64.1% vs. sorafenib 24.3%; *p* = 0.012) after 2 years of follow-up of patients with intermediate-advanced HCC. The BIA by Rognoni et al. [37] reported a greater number of deaths avoided (2 and 14 deaths in 5 and 10 years, respectively) and fewer hospital admissions due to hepatic decompensation (32 hospitalizations avoided in 5 years) in the intermediate-advanced stage. The BIA by Pollock et al. [39] reported an incremental LYG of 0.009 with TARE (1.176 LYG) compared to sorafenib (1.168 LYG) and reported that 71 additional patients would benefit from treatment with curative intent over a 3-year period.

#### Study quality reporting assessment

Approximately six [31, 34–37, 39] of the nine studies (67%) had a high score when evaluated with a 20-items checklist (mean compliance:93%). The remaining three studies (33%) were rated as having a moderate quality (mean compliance: 62%) [32, 33, 38].

## Discussion

This review demonstrates that there is evidence that ^90^Y-TARE is a potentially cost-effective therapy for the treatment of HCC in the intermediate and advanced stages. ^90^Y-TARE was associated with lower treatment costs than sorafenib but higher treatment costs when compared to TACE or ablative therapy. However, the BIA conducted in Canada reflects cost savings associated with ^90^Y-TARE, even when the incremental cost of the device acquisition was considered [32]. Though, studies that compared ^90^Y-TARE with TACE did not account for AEs (postembolization syndrome) [20, 22], a key cost component and lower repetition rate associated with TARE than with TACE [22, 31].

Health outcomes vary with maximum health benefits associated with TARE when compared with TACE for intermediate- [22] and advanced-stage patients [20, 21] and when compared with sorafenib for intermediate- [26] and advanced-stage patients [24–26, 29, 36, 37, 39]. However, the comparison of the effectiveness of TARE versus TACE suggests that TARE may be more beneficial to intermediate HCC as it offers a greater possibility for curative intent in these patients [22]. Similarly, these results suggest that a greater number of patients with advanced HCC can obtain greater clinical benefits from TARE, though at a higher cost [25]. Compared with sorafenib and assuming the same clinical efficacy [24–27, 29, 30], maximum health benefits could be obtained using TARE, given the lower overall cost of TARE reported in studies [24, 25, 27, 29, 30]. Thus, assuming the same health resources for TARE and sorafenib, a greater number of patients could potentially be treated with TARE than with sorafenib, given the cost savings of TARE [32, 37, 39].

Several strengths to our study exist. To our knowledge, this is the first systematic review of the economic evidence of ^90^Y-TARE therapy in hepatic neoplasms that included HCC. This review included a strict inclusion criterion focusing on economic evaluations on TARE in liver neoplasms. An extensive search strategy was conducted by performing a search of both English and Spanish studies from the international bibliographic databases with the largest number of indexed publications (Medline and EMBASE) and of a database of publications in Spanish (MEDES). Also, with the goal of identifying the greatest possible number of studies, communications presented at various international conferences were consulted.

Some limitations to our study exist. First, given English and Spanish studies were included in our review, this may lead to excluding other potential economic evaluations published in other languages. As such, there is a potential for publication bias. Second, the diversity of methodologies used and the different parameters such as a variety of sources of clinical efficacy, comparators, and time horizons may limit the external validity of the results. Third, costs were reported for different dates and currencies, or did not report the reference year for cost items collected. Regardless, costs were adjusted to 2020 ($US PPP costs). Also, studies with missing reference years were assumed to be the same as cost reference sources or the study’s publication year. Fourth, the internal evaluation of the study quality varied as the appraisal of the quality of studies showed considerable differences across studies. Given we included conference abstracts (n = 7) with no full-text version available at the time of this review, this limited the analysis and appraisal of the results. Even though some included studies were abstracts, it is important to note that the results showed similarities with other studies with full manuscripts.

Economic outcomes are dependent on pathology management and affect resource consumption during patient HCC management. The development of new systemic therapies in recent years [41], along with the availability of new diagnostic algorithms for HCC [42], could modify clinical practice guidelines due to earlier detection of the pathology. Another relevant issue is the influence of the radiologist's experience with liver images on determining treatment response [43]. Furthermore, personalised dosimetry with ^90^Y-TARE has shown significant clinical improvement in objective response rate and OS in patients with locally advanced HCC [44]. These parameters are related to resource consumption in clinical practice and may affect the results reported here.

## Conclusion

This review suggests that ^90^Y-TARE contributes to the reduction of hospital resource and therefore reduces costs, improves patient outcomes, and improves the value and efficiency in hospitals. Overall, TARE is a cost-effective short- and long-term treatment for HCC, driven by increased LYG compared to other HCC therapies. Given the evidence highlighted in this review, ^90^Y-TARE is a cost-effective therapy for treating patients with liver neoplasms or HCC in the intermediate and advanced stages. Since clinical practice guidelines or new therapies could potentially impact these results, we recommend future economic evaluations focusing on ^90^Y-TARE from different cost perspectives.

## Supplementary Information

**Additional file 1.** Terminology of searching strategy in PubMed.

## Data Availability

The datasets used and/or analysed during the current study available from the corresponding author on reasonable request. The version contains additional information. The additional information of search strategy is in the Additional file 1.

## Abbreviations

AE: Adverse events
BC: Base case
BCLC: Barcelona Clinic Liver Cancer
BIA: Budget-impact-analysis
CA: Cost-analysis
CEA: Cost-effectiveness-analysis
CHEERS: Consolidated Health Economic Evaluation Reporting Standards
CI: Confidence interval
CIRSE: Cardiovascular and Interventional Radiological Society of Europe
CMA: Cost-minimization-analysis
CT: Clinical trial
CTT: Conventional transarterial therapy
CUA: Cost-utility-analysis
DEB-TACE: Doxorubicin eluting bead transarterial chemoembolization
EANM: European Association of Nuclear Medicine
ECIO: European Conference on Interventional Oncology
ECR: European Congress of Radiology
ESMO: European Society of Medical Oncology
EUNetHTA: European Network for Health Technology Assessment
HCC: Hepatocellular carcinoma
HTA: Health technology assessment
ICER: Incremental cost-effectiveness ratio
ICUR: Incremental cost-utility ratio
ISPOR: International Society for Pharmacoeconomics and Outcomes Research
LMG: Life month gained
LYG: Life years gained
NHS: National Health System
NICE: National Institute for Health and Clinical Excellence
NMB: Net monetary benefit
OECD: Organization for Economic Co-operation and Development
OS: Overall survival
PPP: Purchasing power parity
PRISMA: Preferred Reporting items for Systematic Reviews and Meta-Analyses
QALY: Quality-adjusted life year
REDETS: Network of Health Technology Assessment Agencies
RFA: Radiofrequency ablation
SIO: Society of Interventional Oncology
SNMMI: Society of Nuclear Medicine and Molecular Imaging
SOR: Subgroup or patients with sorafenib
TACE: Transarterial chemoembolization
TAE: Transarterial embolization
TARE: Transarterial radioembolization
TDABC: Time-drive activity-based costing
TS: TARE plus sorafenib
TTP: Time to progression
TTS sequence: TARE, TACE and possibly sorafenib
TKIs: Tyrosine kinase inhibitors
WTP: Willingness-to-pay
^90^Y-TARE: TARE with yttrium 90 microspheres
